# Non-coding RNAs in the interaction between rice and *Meloidogyne graminicola*

**DOI:** 10.1186/s12864-021-07735-7

**Published:** 2021-07-20

**Authors:** Bruno Verstraeten, Mohammad Reza Atighi, Virginia Ruiz-Ferrer, Carolina Escobar, Tim De Meyer, Tina Kyndt

**Affiliations:** 1 Department of Biotechnology, Ghent University, Ghent, Belgium; 2 Department of Environmental Science, University of Castilla-La Mancha, Toledo, Spain; 3 Department of Data Analysis & Mathematical Modelling, Ghent University, Ghent, Belgium

**Keywords:** Non-coding RNAs, Epigenetics, siRNAs, miRNAs, lncRNAs, Nematode, *Oryza sativa*

## Abstract

**Background:**

Root knot nematodes (RKN) are plant parasitic nematodes causing major yield losses of widely consumed food crops such as rice (*Oryza sativa*). Because non-coding RNAs, including small interfering RNAs (siRNA), microRNAs (miRNAs) and long non-coding RNAs (lncRNAs), are key regulators of various plant processes, elucidating their regulation during this interaction may lead to new strategies to improve crop protection. In this study, we aimed to identify and characterize rice siRNAs, miRNAs and lncRNAs responsive to early infection with RKN *Meloidogyne graminicola* (*Mg*), based on sequencing of small RNA, degradome and total RNA libraries from rice gall tissues compared with uninfected root tissues.

**Results:**

We found 425 lncRNAs, 3739 siRNAs and 16 miRNAs to be differentially expressed between both tissues, of which a subset was independently validated with RT-qPCR. Functional prediction of the lncRNAs indicates that a large part of their potential target genes code for serine/threonine protein kinases and transcription factors. Differentially expressed siRNAs have a predominant size of 24 nts, suggesting a role in DNA methylation. Differentially expressed miRNAs are generally downregulated and target transcription factors, which show reduced degradation according to the degradome data.

**Conclusions:**

To our knowledge, this work is the first to focus on small and long non-coding RNAs in the interaction between rice and *Mg*, and provides an overview of rice non-coding RNAs with the potential to be used as a resource for the development of new crop protection strategies.

**Supplementary Information:**

The online version contains supplementary material available at 10.1186/s12864-021-07735-7.

## Background

Rice is one of the most important food crops in the world with an annual worldwide yield of 782 million tons [1]. More than half of the world’s population daily consume rice. Rice is also used as a model species for monocots because of its relatively compact genome and wide array of molecular and genetic resources [2, 3]. Root-knot nematodes are a major pest for rice agriculture. In particular, rice fields infected with root-knot nematode (RKN) *Meloidogyne graminicola* (*Mg*) can show yield losses of up to 70% [4]. They induce giant cells inside the vascular tissue of rice roots, leading to visual symptoms of root galling at the tips [5].

Previous research has shown that components of the epigenetic machinery are differentially expressed during the interaction between rice and *Mg*, such as genes coding for DICER and ARGONAUTE and histone modifying enzymes [6]. Previous work from our lab showed that rice undergoes genome-wide DNA hypomethylation in a CHH context early upon *Mg* infection (3 days post inoculation, 3 dpi), later followed by activation of the corresponding genes. DNA hypomethylation is associated with reduced susceptibility against *Mg,* as shown by experiments with DNA methylation mutants and DNA methylation inhibitor 5-azacytidine [7]. Similarly, we recently demonstrated significant enrichment of acetylation of lysine 9 of histone 3 and trimethylation of lysine 27 of histone 3 as well as significant depletion of dimethylation of lysine 9 of histone 3 in rice galls induced by *Mg* [8].

In this work we focused on the third pillar of epigenetic processes, the role of non-coding RNAs (ncRNAs), in relation to the rice-*Mg* interaction. The ncRNAs are typically grouped in two main classes: small (smRNAs) and long ncRNAs (lncRNAs).

The smRNAs are generally less than 40 nucleotides (nts) in length and are involved in a range of plant physiological responses such as development and stress responses [9–12]. Based on their biogenesis and function, smRNAs can be divided into microRNAs (miRNAs), small interfering RNAs (siRNAs), small nuclear RNAs (snRNAs) and small nucleolar RNAs. Canonically, after transcription, nuclear export and processing, mature miRNAs are short dsRNA segments of which one strand is incorporated in the RISC complex which cleaves or inhibits a complementary target mRNA. MiRNAs play a role in the rice response to a number of (a) biotic stresses such as cold stress and exposure to heavy metals [13–15]. MiRNA osa-miR7695 can enhance resistance against fungal pathogen *Pyricularia oryzae* [16]. Changes in miRNA expression have been described in response to RKN infection in Arabidopsis, tomato, cotton and pea [17–22].

On the other hand, siRNAs derive from dsRNAs replication intermediates or from extensive fold-back structures within virus RNAs [23, 24]. These siRNAs are loaded onto an AGO4 or AGO6 protein, and these complexes are then reimported into the nucleus to target nascent Pol V transcripts still associated with their chromatin template. This leads to the recruitment of DNA methyltransferases that ultimately guide cytosine methylation through RNA-directed DNA methylation (RdDM), mainly targeting transposable elements (TEs) [25, 26]. TEs in promoter regions can affect the expression of the associated gene through DNA methylation changes. Since RdDM mutants show a reduced susceptibility to *Meloidogyne graminicola*, we hypothesized that RdDM related ncRNAs would play a role in the interaction between rice and *Mg* [7]. This hypothesis is strengthened by the observation of several differentially expressed 24 nt-siRNA clusters upon RKN *Meloidogyne incognita* infection in Arabidopsis, which were hypothesized to regulate gene expression via RdDM [27]. Similarly, infection of Arabidopsis with *Meloidogyne javanica* led to a local accumulation of repeat-derived siRNAs [28].

LncRNAs are RNA molecules > 200 nts long with no coding potential. Instead of serving as a template for a functional protein, they seem to execute regulatory roles by at least four different mechanisms: histone and chromatin modifications, transcriptional regulation, target mimicking of miRNAs and post-transcriptional alterations [29]. In *cis*, lncRNAs can influence gene expression negatively or positively. In Arabidopsis, the lncRNAs COLDAIR and COOLAIR - transcribed from the same locus as floral repressor flowering locus C (FLC) - regulate the vernalization process by recruiting the repressive polycomb repressive complex 2 to their locus [30, 31]. *Cis*-acting lncRNAs can also enhance the expression of nearby genes by functioning as enhancers [32].

LncRNAs can also regulate gene expression *in trans* by direct interaction with protein complexes or target mimicry. In *Medicago trunculata* lncRNA *Early nodulin 40* (ENOD40) interacts with RNA-binding protein MtRBP1, which leads to relocalization of MtRBP1 from nuclear speckles to cytoplasmatic granules during nodulation [33]. As target mimics, lncRNAs inhibit miRNA functionality by drawing these molecules away from their true mRNA target [34].

Some lncRNAs have been recently demonstrated to be involved in plant biotic stress responses [35]. Li et al. found 565 lncRNAs to be differentially expressed in tomato after infection with *M. incognita* [36]. Broad insights into the molecular function of lncRNAs in the immune response of rice is lacking however.

In recent years non-coding RNA research has been burgeoning with plenty of promising results for the improvement of crop yield. Overexpression of lncRNA LAIR increases grain yield in rice [37]. In Arabidopsis, silencing of lncRNA ELENA1 increased the susceptibility to *Pseudomonas syringae* pv. tomato DC3000 while overexpression showed the opposite phenotype [38]. Overexpression of lncRNA ALEX1 provided increased resistance against *Xanthomonas oryzae* pv. *oryzae* [39]. Similarly, regulating the expression of small RNAs can provide beneficial effects to plant health: increased expression of miR393 resulted in enhanced bacterial resistance against *Pseudomonas syringae* pv. tomato DC3000 in Arabidopsis [40]. In rice, overexpression of miR397 resulted in an increased grain size and panicle branching [41]. The elucidation of lncRNAs and/or smRNAs with the potential to increase resistance in a globally important crop such as rice against the devastating pest *Mg* would open new avenues for controlling this pathogen.

In this research we aimed to elucidate which small and long non-coding RNAs are affected during the early compatible interaction between rice and the parasitic root-knot nematode *Meloidogyne graminicola* (*Mg*). As time point, we chose for 3 days post inoculation, because this is the earliest time point at which giant cell formation is observable at the rice root tips and because mRNA-seq and DNA methylation data is already available at the exact same time point [7]. Since this nematode typically forms galls at root tips, we collected gall material and compared them with root tips of uninfected plants.

Using high throughput sequencing tools, we generated a comprehensive list of differentially expressed ncRNAs that were used for functional predictions. Independent validation was performed by degradome sequencing and RT-qPCR. This research uncovers ncRNA loci that could play a functional role in the early parasitic interaction between rice roots and *Mg*.

## Results

### Analysis of differentially expressed lncRNAs upon RKN infection in rice

**Fig. 1:**
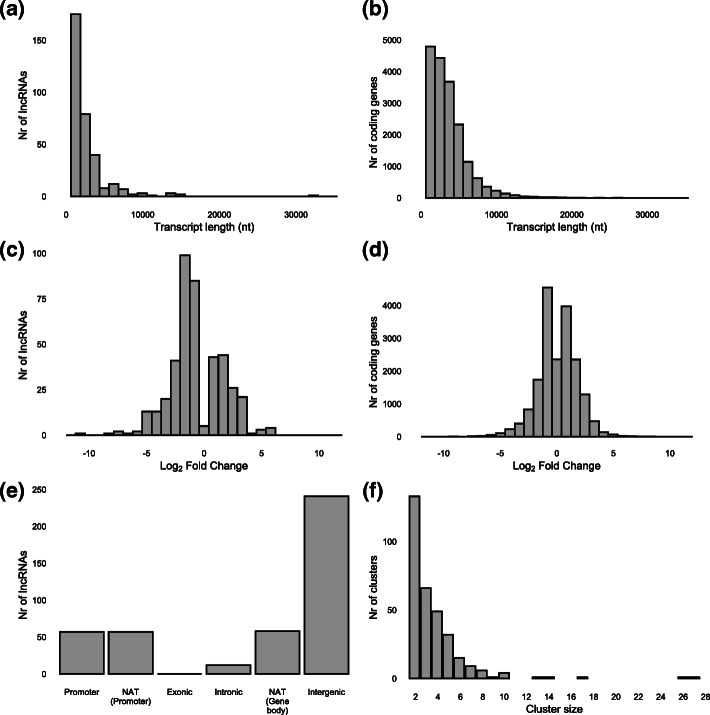
Characteristics of lncRNAs and protein coding genes that are differentially expressed in rice galls induced by *Meloidogyne graminicola* at 3 days post-inoculation. **a** Length distribution of all detected lncRNAs. **b** Length distribution of all detected coding transcripts. **c** Log_2_ Fold Change of differentially expressed (DE) lncRNAs. **d** Log_2_ Fold Change of DE coding transcripts. **e** Genomic positions of DE lncRNAs. **f** Histogram of lncRNA cluster sizes. For (**f**), DE lncRNAs were clustered with upstream/downstream neighbouring coding or non-coding genes that were also DE. The clusters were expanded until no DE coding or non-coding genes were found. Nt: nucleotides, NAT: natural antisense transcript

Total RNA sequencing was executed on 3 biological replicates of 3 dpi galls in comparison with 3 replicates of control root tips in order to identify lncRNAs that are differentially expressed (DE) early after *Mg* infection in rice roots. A total of 223 and 258 million reads were generated for *Mg* and control libraries respectively. After quality control and trimming, 186 million reads of the *Mg* samples and 196 million reads of the control samples mapped uniquely on the rice genome (Supplementary Info File 1). After *Mg* infection, 425 lncRNAs and 18,667 protein coding genes were differentially expressed (DE) in comparison with uninfected root tips (Supplementary Info File 2). The transcripts of DE lncRNAs (median length: 1411) tend to be shorter than DE protein coding genes (median length: 2954) (Fig. 1a & b).

DE lncRNAs tend to be downregulated after *Mg* infection with 66% of DE lncRNAs revealing a negative log2 fold change value in galls versus root tips (Fig. 1c). Protein coding genes DE after *Mg* infection on the other hand have an equal balance of upregulated genes (50%) and downregulated genes (50%) (Fig. 1d).

DE lncRNAs were classified based on their genomic positions. The majority of the DE lncRNAs are intergenic (241/425), 58 DE lncRNAs are natural antisense transcripts (NAT) of gene bodies, 57 lncRNAs overlap with a promoter, 57 lncRNAs are NATs of a promoter region, while 12 lncRNAs overlap with at least one intron. No exonic lncRNAs were found (Fig. 1e).

### Functional prediction of DE lncRNAs

#### Cis activity of lncRNAs

To analyze the potential *cis* activity of DE lncRNAs on neighbouring loci, lncRNA were clustered: “lncRNA-clusters” were created by grouping DE lncRNAs with downstream and upstream neighbouring loci that were also significantly differentially expressed. The clusters were extended until no differentially expressed gene was found further up/downstream. A total of 320 lncRNA clusters was generated (Supplementary Info File 3), encompassing 816 coding and 352 non-coding genes. A majority of these clusters (133/320) consist of two gene members (coding or non-coding), the largest cluster contains 27 gene members (Fig. 1f). To test whether or not DE lncRNAs are enriched in clusters compared to non-DE lncRNAs, we performed a similar procedure by looking upstream and downstream of non-DE lncRNAs for loci that were significantly differentially expressed. 83% (352 of 425) of DE lncRNAs are present in clusters compared to 69% (1614 of 2344) of non-DE lncRNAs, indicating enrichment of DE lncRNAs in these clusters (Chi-square test *P*-value = 7.462e-09).

**Fig. 2:**
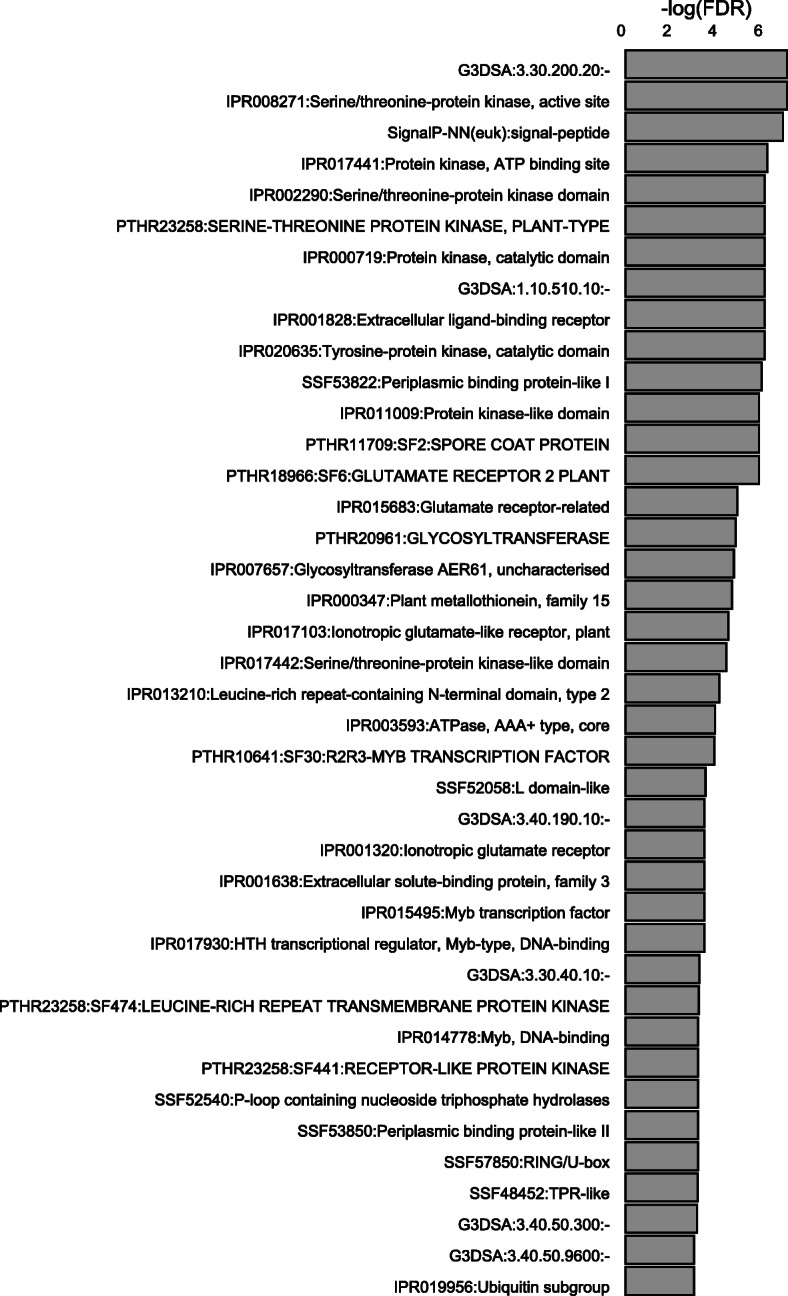
Interaction network between lncRNAs DE after *M. graminicola* infection, miRNAs, and DE protein-coding mRNAs. To build this network, lists of lncRNAs and protein-coding genes that were found to be DE after differential expression analysis were used for miRNA targeting prediction. lncRNAs and protein coding genes that were predicted to be targeted by the same miRNA are shown in the network. The lncRNAs and protein coding genes are shown in red and green respectively. These are indirectly connected through a blue node representing the miRNA that is predicted to target them both. The black arrow points towards miR1850.1 which was found to be DE

Subsequently, we looked at identifying common functionality of coding genes in our clusters by performing enrichment testing for protein domains encoded by these genes. CARMO revealed a total of 40 protein domains to be significantly enriched (FDR < 0.05) amongst those coding genes in the lncRNA clusters (Fig. 2): a.o. kinase domains, specifically serine/threonine kinase domains, leucine-rich repeat (LRR) domains and MYB domains. GO enrichment analysis confirmed enrichment for genes involved in phosphotransferase activity in the lncRNA clusters (Supplementary Info File 4).

Given the known ability of lncRNAs to influence DNA methylation levels *in cis*, the set of differentially methylated regions (DMRs) of Atighi et al. were retrieved to check for colocalization between the detected lncRNA clusters and DMRs [7, 42–44]. Noteworthy, the galls sampled in that study were of exactly the same age and grown under identical conditions as the galls sampled in the current manuscript. These DMRs are genomic regions with a significantly changed DNA methylation pattern between galls and uninfected root tips and are almost exclusively hypomethylated regions (for more details see Atighi et al. [7]). A total of 141 (of 320; 44%) of the here-identified lncRNA clusters show overlap with at least one hypomethylated DMR. Noteworthy, these overlapping clusters contain several significantly upregulated genes with potential ties to the plant immune response, such as MYB transcription factor Os04g0594100 and leucine rich repeat proteins Os07g0498400 and Os05g0406800 (Supplementary Info File 3).

#### Target mimicry activity of lncRNAs

**Fig. 3:**
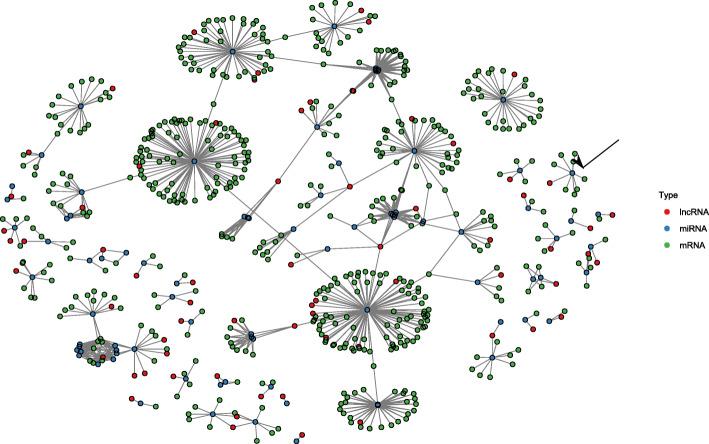
Protein domain analysis of coding genes for which differentially expressed lncRNAs are predicted to serve as target mimics at 3 days post inoculation with *M. graminicola* in rice. The gene IDs of these genes were used as input for the CARMO tool which looks up their annotated protein domains and calculates enrichment

Since lncRNAs can function by acting as target mimics – decoys - to sequester miRNAs away from their true targets, a lncRNA-miRNA-mRNA network was generated. To build this network, we mined for DE lncRNAs that show complementarity with one of the 713 known rice miRNAs available in miRBASE. If such complementarity-based binding between lncRNA and miRNA was predicted, the putative true protein-coding targets of those miRNAs were included in the network. The generated network contains a total of 85 miRNAs (1 DE), 70 DE lncRNAs and DE 529 coding genes (Supplementary Info File 5). Almost all miRNAs in the network are predicted to target multiple coding genes, indicating a potential broad regulatory role for lncRNA target mimics (Fig. 3). One of the miRNAs (osa-miR1850.1) in the interaction network was also found to be significantly upregulated in galls compared to root tips (see further, Table 1).

**Table 1:** Rice miRNAs differentially expressed at 3 days after inoculation with *M. graminicola* as well as their targets according to our degradome sequencing data. Degradome sequencing was performed to evaluate the amount of cleaved miRNA target regions in the investigated tissues. Degradome Log_2_ Fold Change denotes the change in the number of cleaved target fragments in galls versus root tips.’–‘denotes that the degradome data did not indicate significant target cleaving by miRNAs in galls and/or roots

ID miRNA	miRNA Log_2_ Fold change	ID Target	Target Description	Degradome Log_2_ Fold Change
osa-miR398b	−4.48	–	–	–
osa-miR169f.1	−4.17	Os03g0696300	NUCLEAR FACTOR-Y subunit A4	−3.10
		Os03g0174900	NUCLEAR FACTOR-Y subunit A1	−3.67
		Os12g0618600	NUCLEAR FACTOR-Y subunit A10	−1.74
osa-miR397b	−2.27	–	–	–
osa-miR6255	−2.22	–	–	–
osa-miR164a	−2.07	Os04g0460600	NAC domain-containing protein 004	−3.78
osa-miR408-3p	−2.07	Os08g0482700	Cupredoxin domain containing protein	−4.78
osa-miR164d	−1.80	Os04g0460600	NAC domain-containing protein 004	−3.78
osa-miR3979-3p	−1.62	–	–	–
osa-miR166a-3p	−1.33	Os10g0480200	rice homeobox gene 9	0.08
osa-miR166j-3p	−1.21	Os10g0480200	rice homeobox gene 9	0.08
osa-miR167g	−1.15	–	–	–
osa-miR319b	1.62	Os12g0616400	PROLIFERATING CELL FACTOR 8	2.03
osa-miR1850.2	2.02	–	–	–
osa-miR1850.1	2.30	–	–	–
osa-miR5159	2.38	–	–	–
osa-miR319a-3p.2-3p	3.59	Os12g0616400	PROLIFERATING CELL FACTOR 8	2.03

**Fig. 4:**
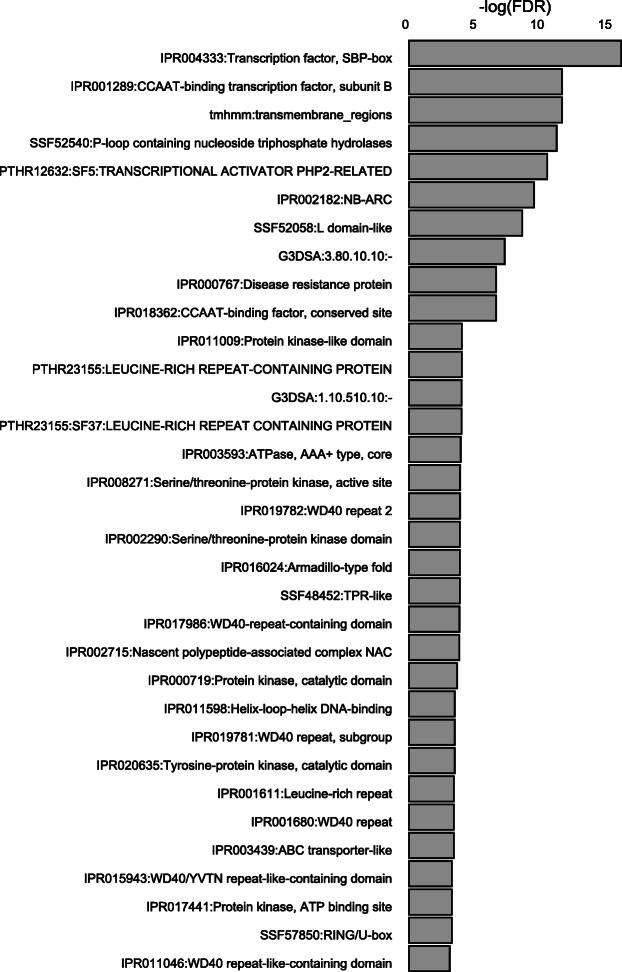
Protein domain enrichment analysis of differentially expressed (DE) coding genes in the identified lncRNA clusters at 3 days post inoculation with *M. graminicola* in rice. The gene IDs of genes neighbouring DE lncRNAs were used as input for the CARMO tool which mines for annotated protein domains and calculates enrichment

The coding genes in the interaction network were mined for enriched protein domains using CARMO (Fig. 4). Interestingly, the obtained results are in concordance with the results of the enrichment testing of coding genes in lncRNA clusters (see Fig. 2), again indicating enrichment for serine/threonine kinase domains and LRRs next to terms like ‘disease resistance protein’, ‘ABC-transporter-like’ and ‘CCAAT-binding transcription factor, subunit B’ or the WD40 domain. GO analysis indicated enrichment for genes with intracellular activity (Supplementary Info File 4).

### Analysis of differentially expressed smRNAs upon RKN infection in rice

A total of 136 million reads and 183 million reads were generated for the small RNA *Mg* and control libraries respectively. After quality control and trimming, 106 million reads of the *Mg* samples and 146 million reads of the control samples mapped uniquely on the rice genome.

### Analysis of expression of miRNAs

We performed differential expression analysis on all 713 annotated miRNAs in rice. A total of 16 miRNAs were found to be significantly differentially expressed (DE) at 3 days after inoculation with *Meloidogyne graminicola* in comparison with root tips of uninfected control plants. Expression of miRNAs is largely suppressed (5 upregulated miRNAs versus 11 downregulated miRNAs (Table 1).

**Fig. 5:**
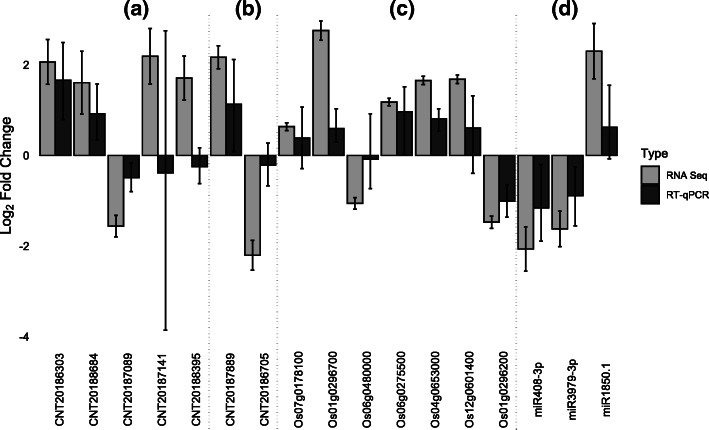
Comparison of RNA Seq and RT-qPCR results of differential expression analysis of a subset of lncRNAs and coding transcripts between 3 dpi galls and uninfected control root tips induced by *M. graminicola* in rice. Error bars represent standard error. **a** lncRNAs predicted to have *in cis* activity. **b** lncRNAs predicted to function *in trans* through miRNA-target mimicry. **c** Protein coding mRNAs potentially affected by this mimicry. **d** miRNAs. Data is based on 3 biological replicates for RNA-seq and 4 biological replicates for RT-qPCR.

Degradome sequencing was performed on a gall and a root tip sample to predict mRNAs targeted by the DE miRNAs (Supplementary Info File 6). A total of 16,333,073 (gall) and 16,652,478 (root tip) raw reads were generated. Targetfinder predicted 642 miRNAs to target at least one mRNA, while overall 5203 mRNAs were predicted to be targeted. In the degradome data we identified mRNAs that were significantly targeted in root and gall samples and this list was matched with the DE miRNAs in these tissues. After filtering, 10 miRNA-target pairs remained. (Table 1). T-plots of these pairs are shown in Supplementary Info File 7. For 8 of these 10 miRNAs-target interactions, the degradome sequencing data agrees with the expected change in expression of the miRNA between galls and root tips, i.e. feature a higher number of cleaved transcripts in galls versus root tips if the miRNA is significantly upregulated in galls versus uninfected root tips or vice versa. The miRNAs for which that pattern does not hold are miR166j-3p and miR166a-3p which both have the same target Os10g0480200, encoding a rice homeobox protein. Interestingly, the miRNAs mainly target transcription factors: miR319b and miR319a-3p.2-3p both target PROLIFERATING CELL FACTOR 8 (PCF8) while miR169f.1 targets three CCAAT-binding transcription factors. miR164d and miR164a both target a NAC domain-containing protein while miR408-3p targets a cupredoxin domain containing protein. Three differentially expressed miRNAs were selected for validation using RT-qPCR, and patterns were in all cases confirmed (Fig. 5).

### Analysis of expression of siRNAs

**Fig. 6:**
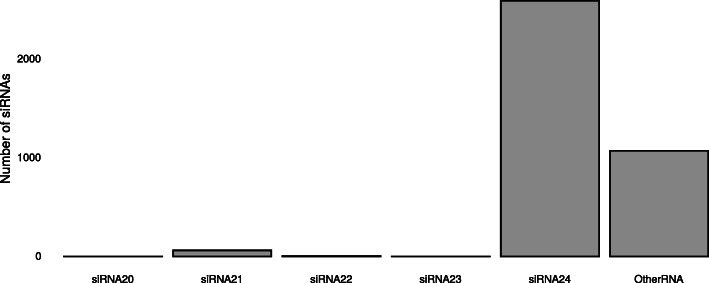
Size distribution of differentially expressed siRNAs 3 days post inoculation with *M. graminicola* in rice. The ‘Other RNA’ class refers to small RNA loci for which no predominant RNA size was found

Differential expression analysis yielded 3739 siRNAs that were differentially expressed after *Mg* infection (Supplementary Info File 8), of which a majority were 24 nts long (Fig. 6). A substantial number of the differentially expressed siRNAs were downregulated (i.e. less present) in galls (58.7%) than upregulated (41.3%).

**Fig. 7:**
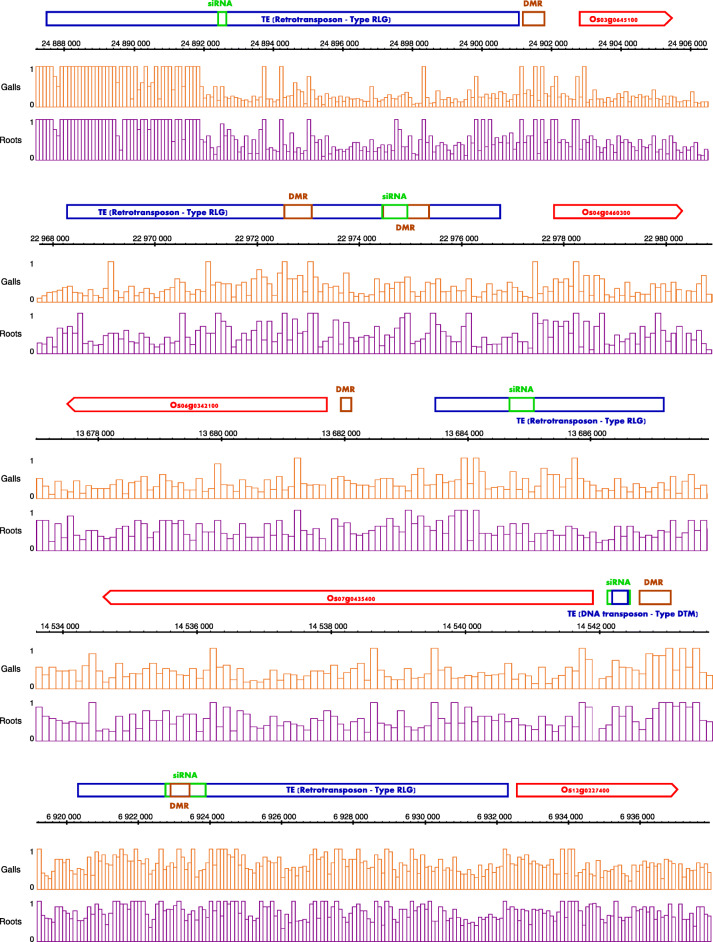
Genomic layout of genes (orange-red arrows) putatively regulated by TE (blue box) based siRNAs (green box) in promoters, based on information from galls at 3 days post inoculation with *M. graminicola*. Orange and purple genome tracks represent CHH DNA methylation percentage in galls and roots respectively. DMR loci (burgundy box) and DNA methylation data were taken from our previously published data [7]. DMR: differentially methylated region; TE: transposable element; siRNA: small interfering RNA locus.

As 24 nt siRNAs often originate from transposable elements (TEs) that can affect the expression of nearby genes through the RdDM pathway, we mined our data for DE siRNA loci that overlap with promoter-based TEs, in other words overlapping with TEs in regions less than 2 kb upstream of the transcription start site of a gene. To narrow down our research for siRNAs with a plausible regulatory role in gene expression, we performed further filtering by requiring that either the TE locus or the genomic region between TE and gene locus overlapped with a DMR in 3 dpi galls [7]. Reduced expression of RdDM related siRNA loci is predicted to lead to reduced DNA methylation [45]. Since genome-wide CHH hypomethylation occurs at this time point after *Mg* infection, we selected for siRNA loci that were downregulated. A final filtering step was performed by selecting downregulated siRNA loci present in a promoter of a significantly upregulated transcript. These filtering steps resulted in a set of 5 genes that are plausible candidates for expression regulation through siRNA activity in their promoter, albeit that one gene has a rather low LFC of 0.27 (Table 2). These genes include a signal recognition particle protein and an allyl alcohol dehydrogenase. The predicted regulatory TE-regions belong to the retrotransposon class RLG (4/5; Class I) or the DNA transposon DTM class (1/5; Class II) of TEs. More details about the expression pattern and genomic locations of the coding genes and associated CHH DMRs, TE and siRNAs can be found in Supplementary Info File 9. A detailed genomic layout of the siRNAs, CHH DMRs, TEs and target genes is shown in Fig. 7.

**Table 2:** Protein coding genes putatively regulated by TE associated siRNAs in their promoters in rice galls at 3 days post inoculation with *M. graminicola*. *LFC* log2 Fold Change, *TE* transposable element

Target	Description	LFC Target gene	siRNA	LFC siRNA
Os03g0645100	Similar to pyruvate dehydrogenase E1 component subunit beta	0.27	osa-b1.0r1–73,430	− 2.14
Os04g0460300	Amino acid transporter, transmembrane domain containing protein	2.04	osa-b1.0r1–86,468	−1.90
Os06g0342100	Signal recognition particle 19 kDa protein (SRP19)	0.76	osa-b1.0r1–108,253	−1.59
Os07g0435400	Similar to WD40	0.50	osa-b1.0r1–120,740	− 2.58
Os12g0227400	Allyl alcohol dehydrogenase	1.66	osa-b1.0r1–41,090	−1.48

To confirm the relevance of the putative siRNA-dependent regulation of gene expression through siRNAs during compatible plant-RKN interactions, we aimed to use a similar strategy with the data from sRNAseq libraries of Arabidopsis galls induced by *M. javanica* versus uninfected roots [20]. As we did for rice, we searched for presence of differentially expressed siRNAs [28] in promoters of genes that were upregulated according to microarray data from microdissected giant cells (GCs) and galls [46]. Ten (GC) and 7 (galls) downregulated siRNAs matched with TEs in promoters of upregulated genes (Table 3). Interestingly, three different siRNAs down regulated in GCs matched a transcriptional repressor of the auxin response inducible (IAA8) involved in lateral root formation, as well as two siRNAs matched a UDP-glucosyl transferase (Table 3) [47]. A beta glucosidase 2 that catalyzes the hydrolysis of the glycosidic bonds to terminal non-reducing residues in beta-D-glucosides and oligosaccharides, a DNA-Methyltransferase MET1 and a transcription factor of the DOF family were also upregulated and coupled with down-regulated siRNAs matching TEs in GCs (Table 3), among others. In galls, TE associated down-regulated siRNAs were detected in the promoter of a Cysteine/Histidine-rich C1 domain family protein, an IQD protein involved in calmodulin-mediated calcium and auxin signaling [48], and a Rhamnogalacturonate lyase family protein, among others (Table 3). Interestingly, in both galls and GCs, most TEs present on these promoter sequences were Class II (mainly HELITRON and MuDR Superfamilies) [28]. More details about the expression pattern and genomic locations of the coding genes and associated TEs and siRNAs can be found in Supplementary Info File 9.

**Table 3:** Protein coding genes putatively regulated by downregulated siRNAs from Arabidopsis galls induced by *M. javanica*, or micro-dissected GCs at 3dpi versus control uninfected tissues, in TEs within their promoters. *LFC* Log2 Fold Change, *TE* transposable element, *GC* giant cells

Target	Description	LFC target gene	siRNA	LFC siRNA
AT1G29980	choice-of-anchor C domain protein, putative (Protein of unknown function, DUF642)	2,42 (GC)	t1095805	−2.15707522
AT1G47655	Dof-type zinc finger domain-containing protein	0,96 (GC)	t0296032	−1.639952
AT2G22670	IAA8: Indoleacetic acid-induced protein 8	1,15 (GC)	t0633011	−1.054989
AT2G22670	IAA8: Indoleacetic acid-induced protein 8	1,15 (GC)	t0018542	−1.303729 S14
AT2G22670	IAA8: Indoleacetic acid-induced protein 8	1,15(GC)	t1235988	−1.0549894
AT3G16520	UDP-glucosyl transferase family protein	2,19(GC)	t0912363	−1.6983221
AT3G16520	UDP-glucosyl transferase family protein	2,19 (GC)	t0001440	−1.09983828
AT5G16580	beta glucosidase 2	0,97 (GC)	t0452534	−1.639952
AT5G49160	MET1	2,09 (GC)	t0659684	−1.05498941
AT5G66050	Wound-responsive family protein	0,92 (GC)	t0006768	−1.153983978
AT1G17480	IQ-domain 7	1,11 (Gall)	t0257580	−2.15707522
AT2G05910	LURP-one-like protein	1,38 (Gall)	t0813649	−1.0549894
AT2G22620	Rhamnogalacturonate lyase family protein (RGIL6)	1,32 (Gall)	t0911003	−1.0549894
AT3G29970	B12D protein	3,31 (Gall)	t0840160	−1.054989
AT4G10265	Wound-responsive protein (WIP3)	1,67 (Gall)	t0092685	−2.03507929
AT4G14980	DC1 domain-containing protein	2,04 (Gall)	t0002719	−1.04626753
AT4G14980	DC1 domain-containing protein	2,04 (Gall)	t1179332	−1.05498941

### Validation of differential expression patterns with RT-qPCR in rice

Seven lncRNAs predicted to be involved in lncRNAs decoy or *in cis* activity were selected for validation with RT-qPCR as well as seven protein coding genes that are present in the identified lncRNA clusters. Finally, three miRNAs were selected for validation. Gene expression trends in RT-qPCR data is in general agreement with gene expression trends from RNA-sequencing data (Fig. 5). Noteworthy, RNA sequencing results showed lower variability and a greater dynamic range compared to the RT-qPCR results.

## Discussion

In this research we aimed to elucidate the characteristics and role of non-coding RNAs in rice in response to *Meloidogyne graminicola* infection. We specifically focused on the early stage of the interaction, 3 dpi, because that is the earliest moment that giant cell formation can be observed in rice. By doing so, we uncover potential early changes that could influence giant cell formation as well as immune responses. Previous transcriptome analysis on this time point has revealed that the immune response of rice is strongly affected at 3 dpi [49]. In addition, DNA methylation analyses revealed that the rice genome is strongly hypomethylated at this time point [7]. Based on the observation that RdDM mutants are significantly less susceptible to *Mg* [7], we hypothesized that non-coding RNAs, which have a major role in this pathway, would also be significantly affected in the rice-*Mg* interaction. That is why we here aimed at uncovering which ncRNAs respond to *Mg* infection at this early time point. Next to functional predictions of lncRNAs and degradome sequencing to confirm miRNA target confirmations, we compared the data with our in-house DNA methylation dataset to detect potential associations between the expression patterns of siRNAs and changes in DNA methylation. The results in this work are a first step in the characterization of non-coding RNA functionality during the infection of rice with *Meloidogyne graminicola* and are therefore largely descriptory, with more research needed to validate our functional predictions. Hence we provide some suggestions for future research along with the discussion of our results.

A total of 425 lncRNAs were found to be differentially expressed when comparing nematode-infected gall tissue with root tips of uninfected control plants. Transcript lengths of DE lncRNAs tend to be shorter than the DE coding genes, which is in agreement with previous lncRNA studies in grape and Arabidopsis [50, 51]. The function of DE lncRNAs was predicted by evaluating two known actions: *in cis* by influencing the expression of nearby genes or *in trans*, as a target decoy, luring away miRNAs from their true target. LncRNAs with a putative *in cis* function were identified by creating clusters that include DE lncRNAs as well as neighbouring DE coding genes, that could hence be regulated by the lncRNA(s). Using this method, 320 lncRNA clusters were found comprising 816 coding genes. To predict target decoy activity, a miRNA-mRNA-lncRNA network was created based on expression changes and target complementarity. Our prediction identified a total of 70 lncRNAs that potentially work as target decoys for 529 coding genes. Protein domain and GO-enrichment analyses revealed that genes in these lncRNA clusters as well as in the miRNA-mRNA-lncRNA network tend to code for proteins containing phosphorylation activity and more specifically contain a serine/threonine kinase domain. Serine/threonine protein kinases are known to be involved in the plant stress response, mainly under abiotic stress, by balancing processes related to growth and plant defense [52, 53]. TaSnRK2.4 an SNF1-type serine/threonine protein kinase in wheat regulates growth, osmotic potential and development [54]. Overexpression of TaSnRK2.4 in Arabidopsis leads to enhanced tolerance against drought, freezing and salt stress. In rice, overexpression of the SNF1-type serine-threonine protein kinase SAPK4 leads to improved germination, growth and development under salt stress [55]. Serine/threonine protein kinases are also involved in biotic stress responses. Overexpression of OsSnRK1a, a regulator of plant primary metabolism, promotes the the salicylic acid pathway and boosts the jasmonate-mediated defense response in rice after inoculation with the blast fungus *Pyricularia oryzae* [56].. Proteins containing a serine/threonine kinase domain can also possess a leucine-rich repeat (LRR) domain, a domain that was also enriched in our protein domain analysis of potential lncRNA target genes. Proteins that contain both domains function as membrane proteins, with the LRR domain being an extracellular receptor of pathogen associated molecular patterns, and the serine/threonine kinase domain intracellularly activating the MAP kinase pathway for defense signaling [57]. LRR domain containing proteins can trigger cell death upon infection [58].

Based on our predictions the here-discovered lncRNAs seem to target – either *in cis* or *in trans* - genes with similar functions, mainly genes coding for serine/threonine protein kinases and transcription factors, which are well known players in the plant immune response. This indicates lncRNAs as a key player in the interplay between rice and *Mg,* although further functional confirmation is needed. Ectopic over-expression of lncRNAs that are predicted to work as a target decoys could confirm their functionality by monitoring transcript and protein levels of the predicted target mRNAs in the generated transgenic lines. The study of lncRNAs with putative *in cis* activity is more challenging, given that their functionality is based on their genomic position. In this case, CRISPR/Cas9 technology could be used with the aim to delete an exon or part of the promoter region [32]. In tomato, CRISPR/Cas9 was successfully used to knock out lncRNA1459 leading to a delay in tomato fruit ripening [59]. Care must be taken however if the lncRNA overlaps with another regulatory element to avoid confounding effects.

Given the ability of lncRNAs to regulate DNA methylation near their own loci [42, 44, 60], we searched for overlap between lncRNA clusters and previously described differentially methylated regions (DMR) in 3 dpi galls and found that 141 of our lncRNA clusters contain a DMR. The fact that these DMRs are almost universally hypomethylated [7] could indicate that upregulated lncRNAs in these DMR-containing clusters negatively regulate DNA methylation, likely through recruitment of demethylases or inhibition of DNA methyltransferases. Downregulated cluster lncRNAs would instead positively regulate DNA methylation through recruitment of DNA methyltransferases or other players in the RdDM pathway [60].

A total of 16 miRNAs were found to be differentially expressed after *Mg* infection. Degradome sequencing revealed cleavage activity for six predicted targets of DE miRNAs corresponding with the change of expression of those miRNAs, most of which were transcription factors. Interestingly, mir169f.1 is indicated to target mRNAs encoding NF-Y subunits, a domain detected to be enriched in the set of coding genes in lncRNA-miRNA-mRNA networks. The miRNA169/NF-Y module is well known to regulate tolerance against biotic and abiotic stresses, both in monocots and dicots. In Arabidopsis, overexpression of miR169 resulted in early flowering [61]. Drought stress in Arabidopsis resulted in downregulation of miR169 and overexpression of its targets conveyed increased resistance to drought [62]. The miR169 family has been shown to be a negative regulator of rice immunity against the fungus *Pyricularia oryzae* [63]. Based on the known negative effects of miR169 expression on rice defense, the here-observed downregulation of miR169 and upregulation of its targets - NF-Y encoding genes - indicate that this module is part of the immune response in rice against *Mg*. Further functional investigation will be needed to prove this hypothesis.

Another downregulated miRNA family after *Mg* infection is the miR164 family, which targets NAC transcription factors, here-confirmed to be less degraded in the investigated tissue. NAC-transcription factors are known regulators of stress responses by initiating cell death, inducing a hypersensitive response or enabling the expression of pathogenesis-related genes [64]. Similar to the mir169/NF-Y module, the miR164/NAC module is also a negative regulator of the plant immune response. Overexpression of miR164 leads to increased rice susceptibility to *Pyricularia oryzae,* while NAC overexpression enhances resistance [65], and has positive effects on drought tolerance and grain yield [66–68]. This indicates that the downregulation of miR169f.1 and the miR164 family in galls, and corresponding accumulation of their target genes are modulating the rice immune response upon nematode infection. Members of the miR319 family were upregulated after *Mg* infection, corresponding with an increased degradation of their target Os12g0616400, encoding a proliferating cell factor (PCF) transcription factor. The miR319/PCF module behaves differently under various stress conditions. Overexpression of miR319 enhances cold tolerance in rice [69, 70], whereas it increases salt and drought tolerance in creeping bentgrass [71]. On the other hand, miR319 acts as a negative regulator of immunity during infection with *Pyricularia oryzae* where expression of miR319 is induced while its targets are suppressed, leading to a negative effect on the JA pathway [72]. MiRNA319-mediated suppression of JA signaling was also observed in rice after infection with rice ragged stunt virus where miR319 was upregulated [73]. Our observation of induced miR319 expression as well as degradation of its targets indicates that there is a similar modulation of the miR319/PCF module after *Mg* infection. Future miRNA research could also focus on the transfer between organisms. Cross-kingdom miRNA transfer has already been described for several pathogens that transfer their miRNAs in order to influence the expression of host genes [74]. However, this transfer can also work the other way: dsRNAs or artificial miRNAs produced by transgenic plants can be taken up by pathogens and have deleterious effects [75]. Recently, multiple miRNAs from wheat Stem Sawfly were described to target wheat mRNAs and vice versa [76]. To investigate this phenomenon in the *Mg*-rice interaction it would be worthwhile to create a protocol to physically separate *Mg* from their galls and sequence them separately, to look for plant and nematode miRNAs respectively.

Finally, a total of 3739 siRNAs were found to be DE in rice after *Mg* infection, most of which were 24 nts long, with slightly more siRNAs downregulated than upregulated. The observation that 24 nt siRNA are most responsive to nematode infection is in agreement with previous findings that highlighted an enrichment of 24 nt sequences in Arabidopsis roots infected with *Meloidogyne javanica* or with *M. incognita* versus control plants [20, 28]. Twenty-four nt-siRNAs tend to originate from transposable elements and are involved in *in cis* DNA methylation, e.g. by presence in promoter regions [77]. Our research uncovered five coding genes that are potentially regulated by siRNA-containing promoters. These genes include Os12g0227400, an allyl alcohol dehydrogenase (ADH) gene that is upregulated in galls. In Arabidopsis, ADH1 expression was induced upon salt and drought stress as well as upon infection with *Pst* DC3000, resulting in increased accumulation of callose deposition and soluble sugars [78]. Overexpression of ADH1 increased biotic and abiotic stress resistance. Further research using an overexpression line for example could elucidate if enhanced ADH expression would also confer beneficial effects to the rice immune response in the rice-*Mg* interaction.

Interestingly, these analyses showed that most of the induced genes potentially regulated by siRNAs matching TE-containing promoters, encode proteins involved in related biological processes in both rice-*M. graminicola* and Arabidopsis-*M. javanica* nematode interactions (Tables 2 and 3 respectively). Among them, genes involved in carbohydrate metabolism (a rice pyruvate dehydrogenase E1 component subunit beta and an Arabidopsis beta glucosidase 2), other metabolic genes as for example an allyl alcohol dehydrogenase coding gene in rice or an UDP-glucosyl transferase in Arabidopsis, as well as proteins containing DNA-binding domains (WD40 in rice and MET1 a DC1 domain-containing protein and a Dof-type zinc finger domain-containing protein from Arabidopsis) (Tables 2 and 3). The resemblances in the putative cell functions of these siRNAs targeted genes detected in the RKN-interactions either with a monocotyledonous or a dicotyledonous plant species, suggest the importance of siRNAs gene regulation in at least part of the crucial basic cellular processes leading to a compatible plant-nematode interaction. Overexpression lines of siRNAs can help with further characterization of the regulatory effects of these siRNAs.

## Conclusions

In all, smRNAs and lncRNAs differentially expressed after *Meloidogyne graminicola* infection appear to target many genes with critical roles during the plant stress response, which in turn indicates their potential importance in regulation of the rice immune system. Differentially expressed miRNAs were mostly downregulated and their targets, which were confirmed using degradome sequencing, are primarily transcription factors known to be involved in plant stress responses. Small RNA results compared with DNA methylation data led to the discovery of promoter-based siRNAs putatively regulating gene expression. We predicted the *in cis* functionality of differentially expressed lncRNAs by identifying adjacent putative target genes and made lncRNA-miRNA-mRNA networks to study the possibility of lncRNAs to serve as target decoys. Both approaches identified genes coding for serine/threonine kinases as the main putative lncRNA targets. This work provides new insights in the behavior of ncRNAs in rice during *Mg* infection. Further functional characterization of these ncRNAs will help to reveal how this information can help to design effective plant protection strategies against parasitic nematodes.

## Methods

### Plant growth, nematode inoculation and sampling of material

*Oryza sativa* L. cv ‘Nipponbare’ (GSOR-100, USDA) seeds were germinated for 5 days at 32 °C on tissue drenched with distilled water. Seedlings were transferred into SAP substrate (sand-absorbent polymer) and grown at 28 °C under a 16 h/8 h light/dark regime. After 2 weeks, plants were inoculated with ca. Five hundred stage two juveniles of root-knot nematode *Meloidogyne graminicola (Mg)* per set of three plants. Control plants were mock-inoculated with water. After 36 h, plants were transferred to 50% Hoagland solution in glass tubes to synchronize infection. Root tips of uninfected plants are the ideal control material for interaction studies between rice and *Mg*, as these nematode typically infect root tips [5]. Root tips and galls were collected from control and inoculated plants respectively, 3 days post inoculation (3 dpi). This is the earliest time point at which formation of giant cells can be observed in rice and allows for comparison with our previously generated DNA methylation dataset from 3 dpi galls [5, 7]. Three biological replicates were sampled per condition, each biological replicate being derived from a pool of ca. Nine plants to reduce variance not associated with the treatment, and flash-frozen in liquid nitrogen.

### RNA extraction and smRNA sequencing

Frozen samples were ground and total RNA was extracted using the ZR Plant RNA Miniprep kit (Zymo Research). The bead beater step was performed using a speed of 4 m/s for 45 s. All centrifugal steps were performed at 16000 x g.

For DNase treatment 1 μg of total RNA per sample was combined with 3.6 μL DNase I buffer + MgCl_2_ (B43, Thermo Fisher), 1 μL RiboLock RNase Inhibitor (EO0381, Thermo Fisher) and 1 μL DNase I (EN0521, Thermo Fisher). RNase-free water was added until a total volume of 36 μL was reached followed by incubation for 30 min at 37 °C. Finally, 4 μL 25 mM EDTA was added before incubating for 10 min at 65 °C.

Quality of total RNA of gall and control samples was verified with an RNA 6000 p chip (Agilent technologies) and concentrations were measured with a Quant-it Ribogreen RNA assay (Life technologies). Three hundred fifty ng of RNA was used with the Small RNA seq library prep for Illumina (Lexogen) with the small RNA dual indexing (Lexogen). Library prep was performed according to the manufacturer’s instructions. Briefly, 3′ adapters were ligated and excess 3′ adapters were removed. 5′ adapters were then ligated, followed by cDNA synthesis of the ligated RNA. The cDNA was used for enrichment PCR (14 cycles) and purified with AMPure XP purification (1:1.8) (Beckman Coulter). Adapter dimers were removed using an 8% native PAGE gel (Life Technologies). The quality was checked with a High sensitivity DNA chip (Agilent technologies). Quantification of the libraries was performed with a qPCR assay according to Illumina protocol to enable equimolar pooling of libraries. An additional purification step was performed on E-gel EX agarose gel (2% agarose). Finally, sequencing was performed on a Hiseq 3000 using 5% Phix spike-in (single end reads, 50 bp).

### Data analysis of small RNA

Quality control was performed using FastQC (version 0.11.8) [79]. Trimming was performed using Trimmomatic (version 0.38) setting following parameters: ILLUMINACLIP:3:30:10, MAXINFO:23:1, SLIDINGWINDOW:5:20, MINLEN:17 [80]. STAR (version 2.6.1d) was used for mapping with parameter settings for small RNA mapping as used for small RNA analysis within the ENCODE project [81]: readFilesCommand zcat, outFilterMismatchNoverLmax 0.05, outFilterMatchNmin 16, outFilterScoreMinOverLread 0, outFilterMatchNminOverLread 0, alignIntronMax 1, outSAMmultNmax 1 and runRNGseed 777 [82]. Raw and trimmed read counts and mapping statistics can be found in Supplementary Info File 1.

### miRNA analysis

For differential expression analysis, a GTF file was downloaded from miRBase (version 21) with all known mature miRNas in rice [83]. Following R packages were then used to create a count table with read counts: rtracklayer (version 1.44.4) to convert the GTF file into a Granges object [84], Rsamtools (version 2.0.3) to create a BamFileList object [85] and GenomicAlignments (version 1.20.1) for the summarizeOverlaps function to create the count table [86]. The following options were used for counting: mode = ‘Union’ and singleEnd = TRUE. The DESeq2 package (version 1.24) was used to detect significant differences between conditions at a Benjamini-Hochberg false discovery rate (FDR) of 0.05 [87]. Control samples were set as reference for log fold change calculations.

### Degradome sequencing and data analysis

Degradome sequencing was performed on a single 3 dpi gall sample and single uninfected rice root tip sample. Plant growth and treatment conditions, RNA extraction and DNAse treatment were as described in previous paragraphs. Samples were sent to LC Sciences (Houston, Texas, USA) where total RNA was extracted using Trizol reagent (Invitrogen, CA, USA) following the manufacturer’s procedure. Total RNA quality and quantity were analysed with a Bioanalyzer 2100 and RNA 6000 Nano Lab Chip Kit (Agilent, CA, USA) with RIN number > 7.0. Approximately 20 μg of total RNA was used to prepare the degradome library. Approximately 150 ng of poly(A) + RNA was used as input RNA and annealed with biotinylated random primers, followed by streptavidin capture of RNA fragments through the biotinylated random primers. 5′ adaptors were ligated exclusively to RNAs containing 5′-monophosphates. Reverse transcription and PCR libraries were sequenced using the 5’adapter only, resulting in the sequencing of the first 36 nucleotides of the inserts that represented the 5′ ends of the original RNAs. Finally, single end 50 bp sequencing was performed on an Illumina Hiseq 2500.

Bioinformatic analysis was performed using the ACGT101-DGD pipeline (LC Sciences, version 3.1). Raw sequencing reads were obtained using Cutadapt (version 1.10) perl scripts to remove adaptors and low quality reads [88]. The extracted sequenced reads were then used to identify potentially cleaved targets by the CleaveLand pipeline [89]. More specifically, degradome reads were mapped to the mRNA sequences downloaded from the genome database (Ensembl, version 37). Only perfectly matching alignment(s) for the given read were kept for degradation analysis. All resulting reads (“t-signature”) were reverse-complemented and aligned to the miRNA identified in our study using miRBase. Alignments where the degradome sequence position coincided with the tenth or eleventh nucleotide of miRNA were retained and scored. In addition, to easily analyze the miRNA targets and RNA degradation patterns, t-plots were built according to the distribution of signatures (and abundances) along these transcripts.

### Data analysis of lncRNAs

Total RNA sequencing data (GSE152783) was generated in our recent study on histone modification dynamics upon RKN infection in rice [8], in which samples (3 biological replicates of 3 dpi galls and 3 uninfected root tips) were collected under exactly the same conditions as described above. In that previous study, only mRNAs were used, in order to compare gene expression patterns with histone modification patterns. Here, we focus in detail on the non-coding transcripts. The quality of the reads was verified with FastQC (version 0.11.8) [79]. Reads were trimmed using Trimmomatic (version 0.38) with following parameters: ILLUMINACLIP:3:30:10, MAXINFO:23:1, SLIDINGWINDOW:5:30, MINLEN:17 [80]. STAR (version 2.6.1d) was used for mapping with the following parameters: readFilesCommand zcat, outFilterMultimapNmax 1 and outSAMtype BAM SortedByCoordinate. Afterwards, samtools (version 1.10) was used to merge multiplexed samples [90]. Raw and filtered read counts, and mapping statistics can be found in Supplementary Info File 1.

GTF files were acquired from the CANTATA2 database containing all lncRNAs annotated in rice and combined with those from Ensembl (release 42) [91, 92]. Counts of alternative transcripts were combined. A count table was made using the following R packages: rtracklayer (version 1.44.4) to convert the GTF file into a Granges object [84], Rsamtools (version 2.0.3) to create a BamFileList object [85] and GenomicAlignments (version 1.20.1) for the summarizeOverlaps function to create the count table [86]. The following options were used for counting: mode = ‘Union’, preprocess.reads = invertStrand, singleEnd = TRUE and ignore.strand = FALSE. To find differentially expressed transcripts/RNAs between galls and uninfected root tips, DESeq2 (version 1.24.0) was used with a Benjamini-Hochberg FDR cutoff of 0.05.

*Cis* activity of lncRNAs was predicted by clustering differentially expressed lncRNAs with neighbouring genes that are also differentially expressed in the rice-RKN interaction. Clusters were expanded upstream and downstream until no differentially expressed genes were detected. Transcripts that were discarded by independent filtering by DESeq2 were considered to be not differentially expressed. Associations between lncRNA activity and DNA methylation changes were analyzed by comparisons with differentially methylated regions (DMRs) between 3 dpi galls and roots identified in Atighi et al. [7].

The potential of lncRNAs to function as target mimics for coding transcripts was evaluated by psRNAtarget. All sequences of diferentially expressed lncRNAs were entered in psRNAtarget to identify miRNAs that may target differentially expressed lncRNAs (expectation value > 3.0) [93]. After retrieving their sequences from miRBase (version 21), these miRNA sequences were entered in psRNAtarget to find putative mRNA targets (expectation value > 3.0) [83]. Triplets of mRNA-miRNA-lncRNA were filtered using two conditions: (1) the expression pattern of the lncRNA reflects that of the mRNA, because upregulation of a decoy lncRNA is assumed to lead to reduced targeting of the corresponding mRNA, and hence over-accumulation; (2) the mRNA is significantly differentially expressed between galls and uninfected root tips.

Enrichment of protein domains annotated by Interproscan was assessed using CARMO [94, 95]. Gene ontology (GO) enrichment was evaluated with AgriGO2 [96].

### RT-qPCR validation

Validation of ncRNA expression was performed with RT-qPCR on independent samples, taken from plants grown and treated as described above. Four biological replicates, each consisting of a pool of 8–9 plants, were collected per treatment. RNA extraction, cDNA synthesis and RT-qPCR was performed as described in Singh et al. [97]. All primers were manufactured by Sigma-Aldrich (United Kingdom) (Supplementary Info File 10).

For RT-qPCR on miRNAs the stem-loop PCR method of Varkonyi-Gasic et al. (2007) was used [98]. For normalization, miR390-5p and miR7694-3p were used, because they reveal stable expression in the investigated material [99].

Two technical replicates were used per PCR reaction. A no template control was included as negative control. CFX Manager (version 3.1.1217.0823) was used for qPCR analysis in the regression modus. Quality control and filtering was performed based on melting peak analysis. Normalization and differential expression were analyzed using the REST 2009 software [100].

### siRNA analysis

First, a merged annotation file was created, combining the rice information of two siRNA databases: the plantsmallrnagenes database and the Pln24NT database [101, 102]. The differential expression pipeline was executed as described above for miRNAs except that the fdrtool R package was used for local FDR adjustment [103].

A selection of siRNAs was made based on overlap with transposable elements (TEs), as listed in the rice TE database [104]. These siRNAs were then further filtered, based on overlap with promoter regions, defined as the 2 kb region upstream of a transcription start site. The association between siRNA activity and DNA methylation changes was analyzed. A list of differentially methylated regions (DMRs) between 3 dpi galls and roots was obtained from Atighi et al., 2020 [7]. DMRs that overlapped with the same promoters as the selected siRNAs were retained.

In Arabidopsis, we selected siRNAs from six independent sRNAs libraries, three from galls induced by *M. javanica* at 3dpi and three from control root samples (GSE71563) [20], that fully match repetitive Arabidopsis DNA sequences deposited in the repetitive elements database Repbase (rasiRNAs) [105]. Reads were cleaned as described previously [20]. Count data of rasiRNAs were normalized with respect to the total number of sRNAs per sample. We used the stats package of R [106] to perform Student’s t-test analysis (rowtest) after which *P*-values were adjusted by a Benjamini-Hochberg FDR correction. Genes that are upregulated in galls and micro-dissected giant cells (GCs) compared to their corresponding control tissues were selected based on microarrays performed in Arabidopsis at 3 dpi [46]. Unique differentially expressed rasiRNAs with 100% sequence homology to the promoter sequence of those selected differentially expressed genes were identified. Further analysis focused on promoters containing TEs that differentially accumulate rasiRNAs in galls as compared to control roots. TEs were further classified as Class I (retrotransposon) or Class II (DNA transposon) as well as classified their family and superfamily. For genome annotation of genes, promoters, TEs and significant rasiRNAs the TAIR10 database was used.

## Supplementary Information

**Additional file 1.** Read counts of small and total RNA libraries (raw, after trimming and after mapping). Table S1 read counts of total RNA libraries. Table S2 Read counts of small RNA libraries.

**Additional file 2.** Results of differential expression analysis on total RNA data. Table S3 Statistics of differential expression analysis.

**Additional file 3.** List of lncRNA clusters and their overlap with differentially methylated regions. Table S4 Expression and genomic location of lncRNA clusters.

**Additional file 4.** Results of gene ontology analysis on genes in lncRNA clusters and genes predicted to be involved in lncRNA mimicry. Table S5 All significantly enriched GO terms amongst protein coding genes in lncRNA clusters. Table S6 All significantly enriched GO terms amongst protein coding genes that are predicted to be involved in a lncRNA decoy - miRNA - mRNA triplicate.

**Additional file 5.** List of lncRNAs and mRNAs predicted predicted to be involved in lncRNA mimicry. Table S7 Mirnas with predicted target mRNA or decoy lncRNA.

**Additional file 6.** Degradome sequencing results. Table S8 Degradome statistics and miRNA annotation.

**Additional file 7.** T-plots of differentially expressed miRNAs. Fig. S1-S10 T-plots of differentially expressed miRNAs in gall and root tip samples.

**Additional file 8.** SiRNAs found to be differentially expressed in rice after *Meloidogyne graminicola* infection. Table S9 Statistics of differential expression analysis and genomic locations of differentially expressed siRNAs.

**Additional file 9.** Details of siRNAs predicted to regulate gene expression via the promoter region. Table S10 Expression and genomic location data about siRNAs predicted to regulate gene expression via the promoter region in the rice - *M.graminicola* interaction. Table S11 Expression and genomic location data about siRNAs predicted to regulate gene expression via the promoter region in the Arabidopsis - *M. javanica* interaction using giant cell (3 dpi) data. Table S12 Expression and genomic location data about siRNAs predicted to regulate gene expression via the promoter region in the Arabidopsis - *M. javanica* interaction using gall (3 dpi) data.

**Additional file 10.** List of primers used in this study. Table S13 Forward and reverse primers.

## Data Availability

The datasets supporting the conclusions of this article are available in the Gene Expression Omnibus repository. Small RNA data generated in this study were deposited under accession no. GSE156244 (https://www.ncbi.nlm.nih.gov/geo/query/acc.cgi?acc=GSE156244). Small RNA data of Arabidopsis was already available (accession no. GSE71563, https://www.ncbi.nlm.nih.gov/geo/query/acc.cgi?acc=GSE71563). Total RNA data and DNA methylation data used in this study are available under accession no. GSE152783 (https://www.ncbi.nlm.nih.gov/geo/query/acc.cgi?acc=GSE152783) and GSE130064 (https://www.ncbi.nlm.nih.gov/geo/query/acc.cgi?acc=GSE130064) respectively.

## Abbreviations

DE: Differentially expressed
DMR: Differentially methylated region
GC: Giant cell
GO: Gene Ontology
LFC: Log_2_ fold change
lncRNA: Long non-coding RNA
*Mg*: *Meloidogyne graminicola*
miRNA: microRNA
NAT: Natural antisense transcript
ncRNA: Non-coding RNA
nt: Nucleotide
RdDM: RNA-directed DNA methylation
RKN: Root-knot nematode
RT-qPCR: Reverse transcriptase quantitative polymerase chain reaction
siRNA: Small interfering RNA
smRNA: Small RNA
snRNA: Small nuclear RNA
TE: Transposable element
