# Correction to: Non-coding RNAs in the interaction between rice and *Meloidogyne graminicola*

**DOI:** 10.1186/s12864-022-08342-w

**Published:** 2022-02-07

**Authors:** Bruno Verstraeten, Mohammad Reza Atighi, Virginia Ruiz-Ferrer, Carolina Escobar, Tim De Meyer, Tina Kyndt

**Affiliations:** 1 Department of Biotechnology, Ghent University, Ghent, Belgium; 2 Department of Environmental Science, University of Castilla-La Mancha, Toledo, Spain; 3 Department of Data Analysis & Mathematical Modelling, Ghent University, Ghent, Belgium

**Correction to: BMC Genomics 22, 560 (2021)**

**https://doi.org/10.1186/s12864-021-07735-7**

Following publication of the original article [1], it was reported that the Funding declaration was missing a grant. The updated Funding declaration is given below with the previously missing information given in boldface:

This work was supported by Fonds Wetenschappelijk Onderzoek (FWO)- Vlaanderen (G007417N), Bijzonder Onderzoeksfonds UGent (BOF-starting grant to TK) **and by The Spanish Ministry of Science and Innovation, PID2019-105924RB-I00 (MCIN/ AEI /10.13039/501100011033 to CE)**. MRA was supported by Ministry of Science and Technology of Iran.

The original article [1] has been corrected.
