# Supplementary material for: Non-coding RNAs in the interaction between rice and Meloidogyne graminicola
Source: BMC Genomics. 2021 Jul 20;22:560. doi: 10.1186/s12864-021-07735-7 (PMC8293575; doi:10.1186/s12864-021-07735-7)
Supplement: Supplementary file 7 — Additional file 7. T-plots of differentially expressed miRNAs. Fig. S1-S10 T-plots of differentially expressed miRNAs in gall and root tip samples. [file 12864_2021_7735_MOESM7_ESM.pdf]

Supplementary Info File 7 T-plots of differentially expressed miRNAs.

For details about alignment score, category number and *P*-value calculation, see the manual of the Cleaveland pipeline and the ACGT101-DGD pipeline of LC Sciences.

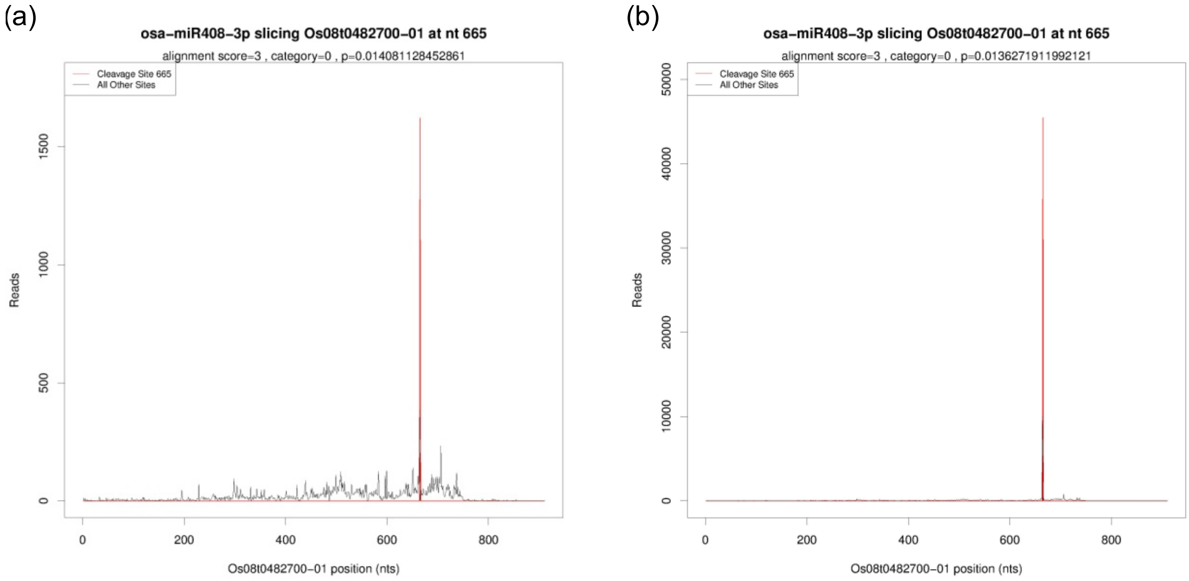

Figure S1 T-plots of miR408-3p targeting Os08t0482700-01 in (a) gall sample; (b) root tip sample.

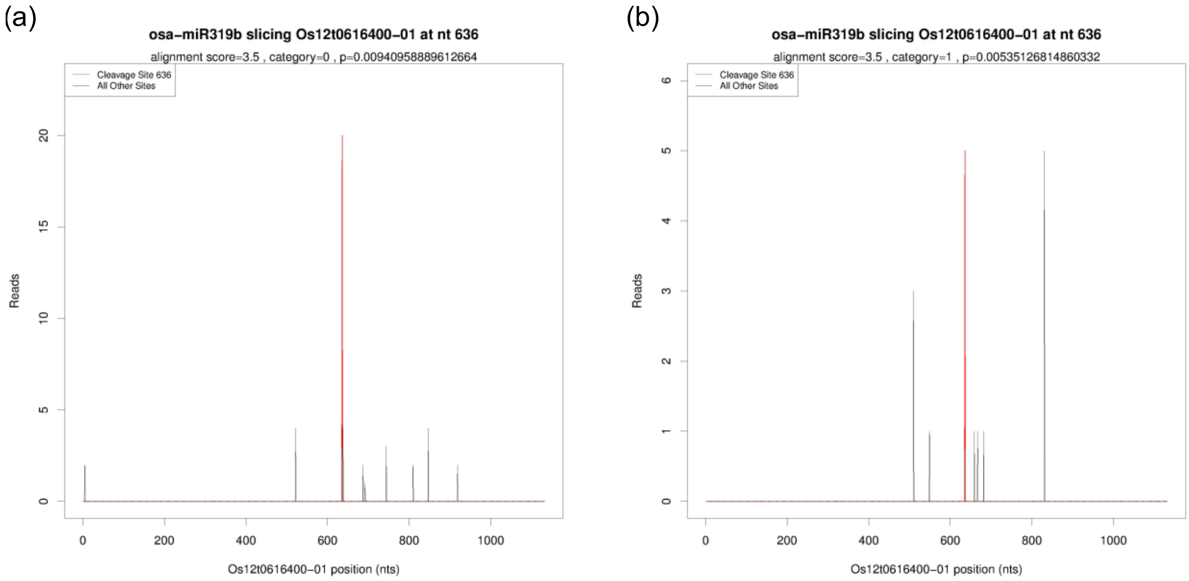

Figure S2 T-plots of miR319b targeting Os12t0616400-01 in (a) gall sample; (b) root tip sample.

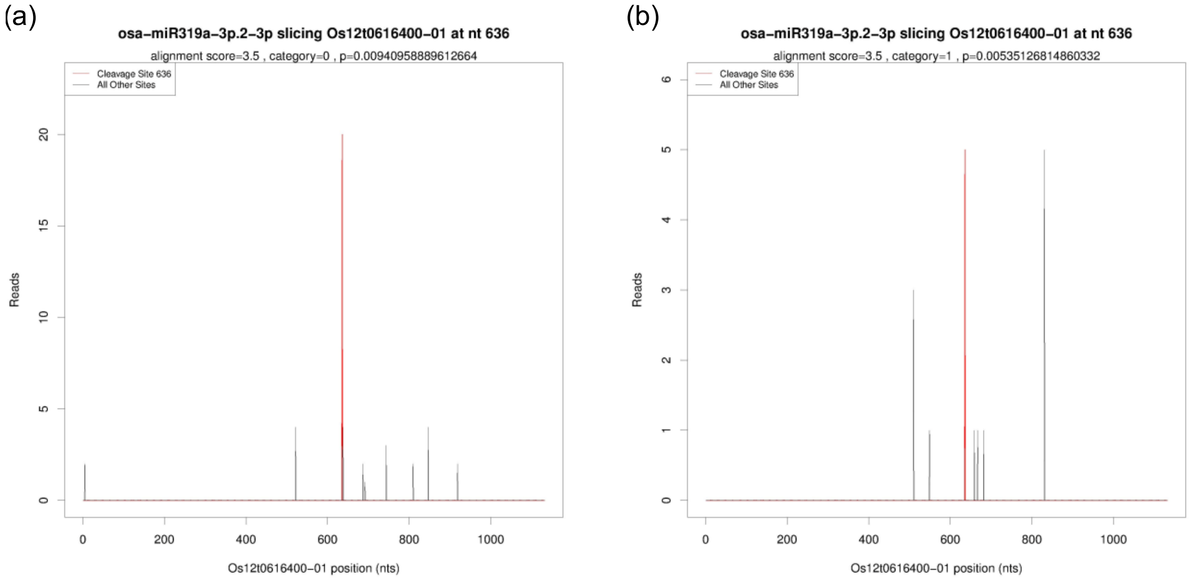

Figure S3 T-plots of miR319a-3p.2-3p targeting Os12t0616400-01 in (a) gall sample; (b) root tip sample.

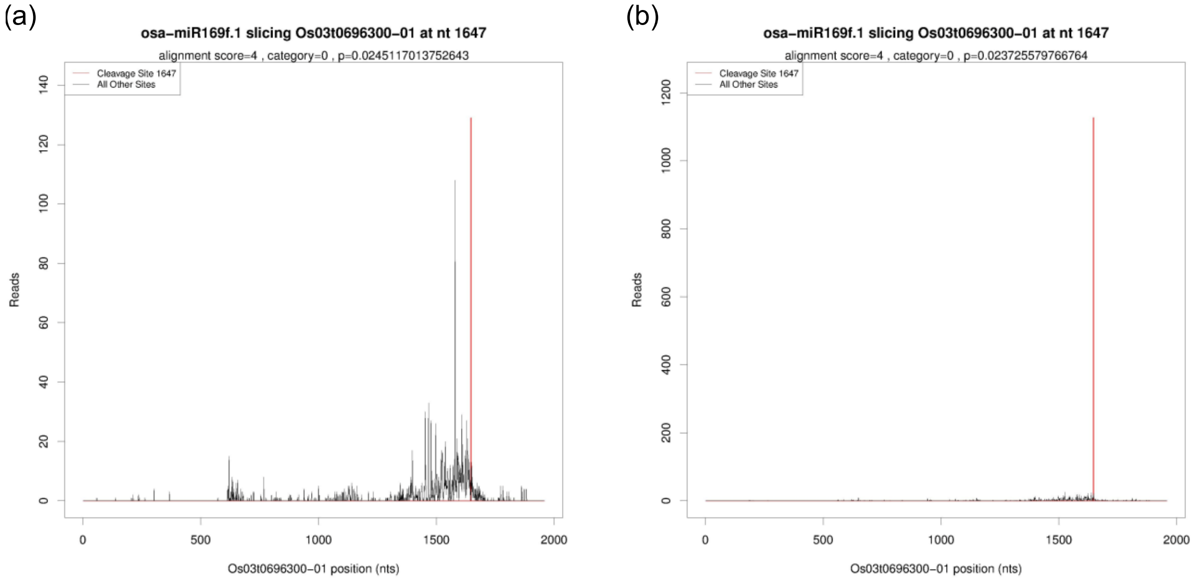

Figure S4 T-plots of miR169f.1 targeting Os03t0696300-01 in (a) gall sample; (b) root tip sample.

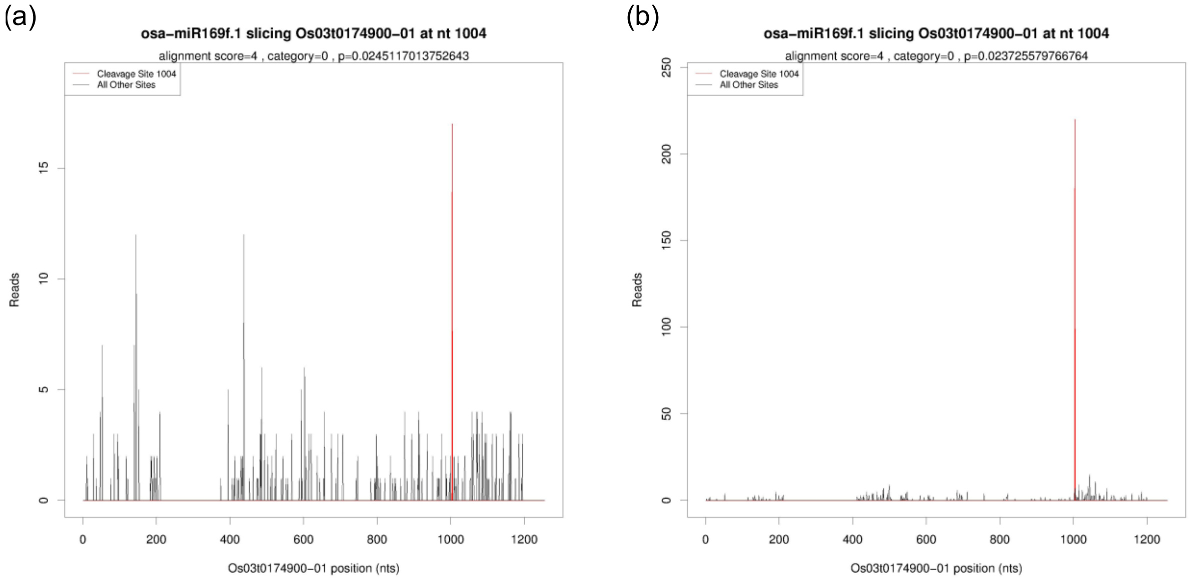

Figure S5 T-plots of miR169f.1 targeting Os03t0174900-01 in (a) gall sample; (b) root tip sample.

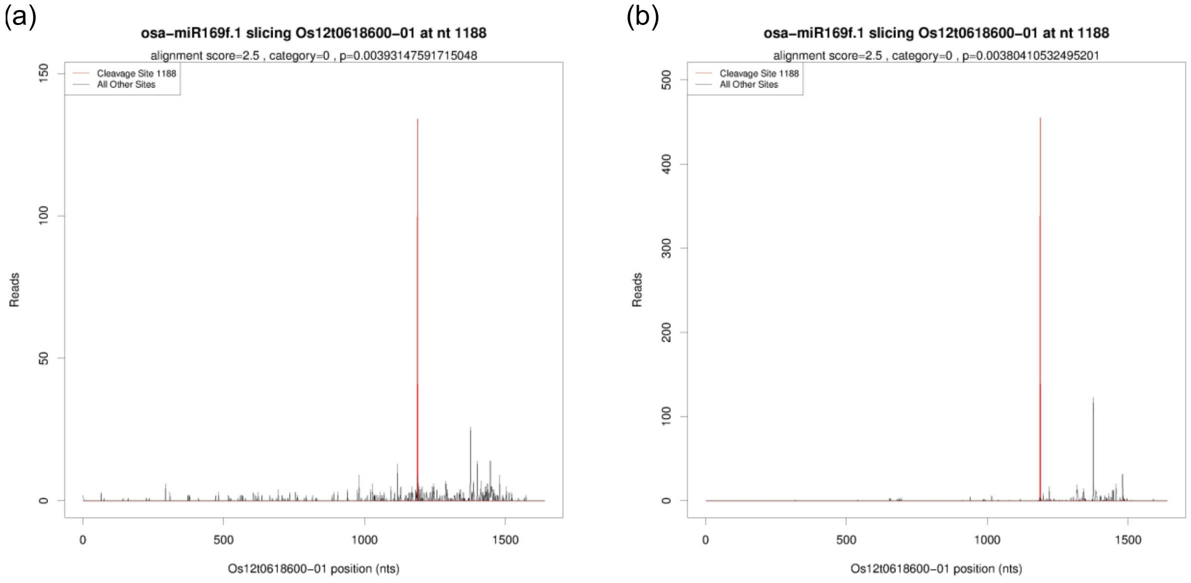

Figure S6 T-plots of miR169f.1 targeting Os12t0618600-01 in (a) gall sample; (b) root tip sample.

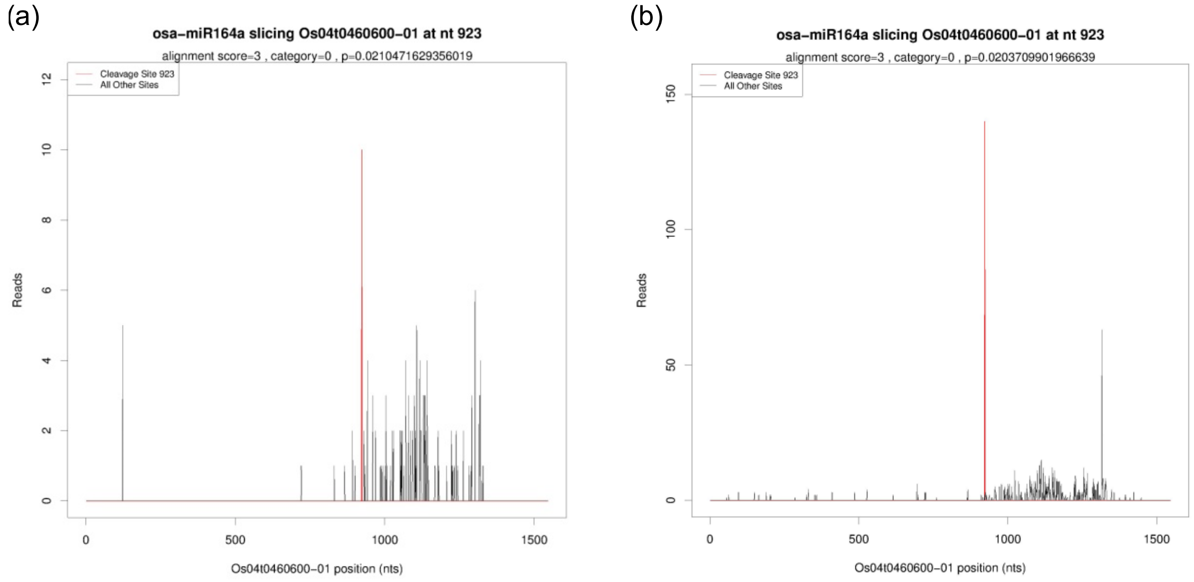

Figure S7 T-plots of miR164a targeting Os04t0460600-01 in (a) gall sample; (b) root tip sample.

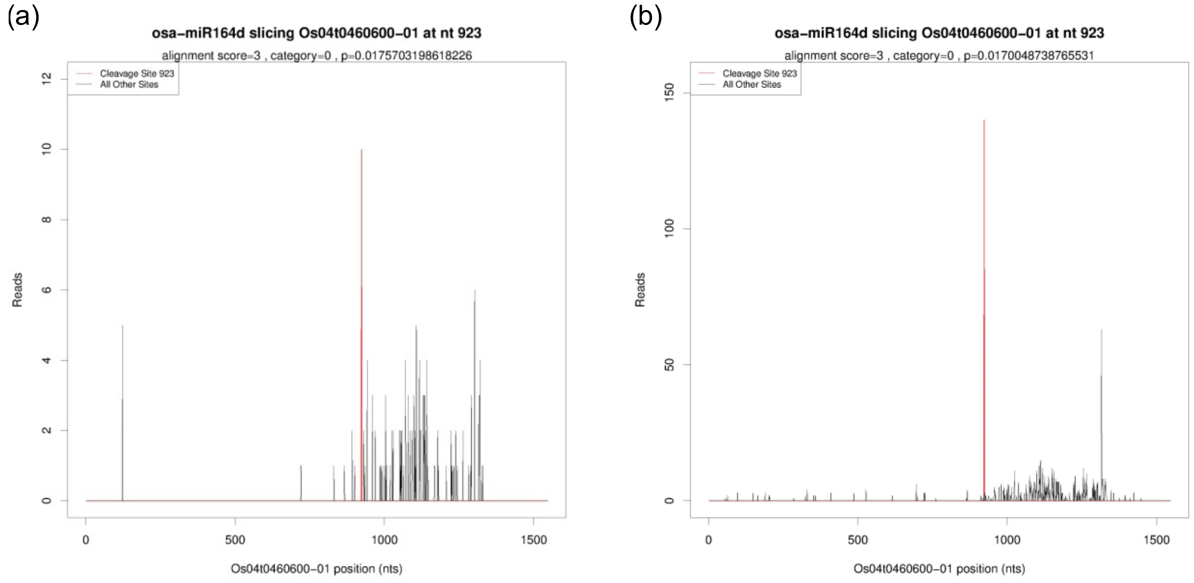

Figure S8 T-plots of miR164d targeting Os04t0460600-01 in (a) gall sample; (b) root tip sample.

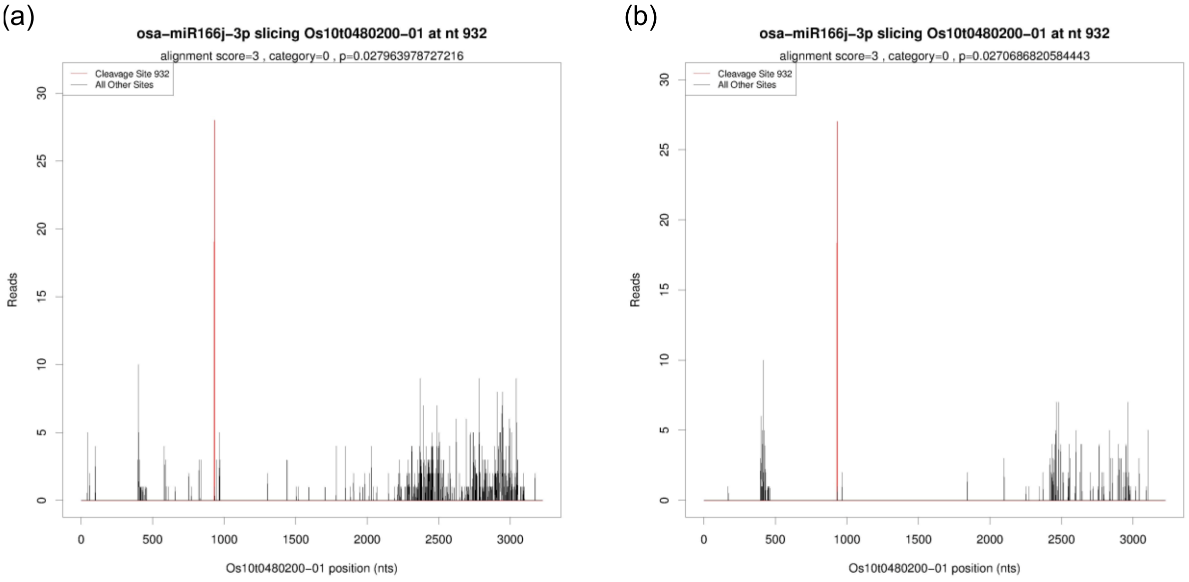

Figure S9 T-plots of miR166j-3p targeting Os10t0480200-01 in (a) gall sample; (b) root tip sample.

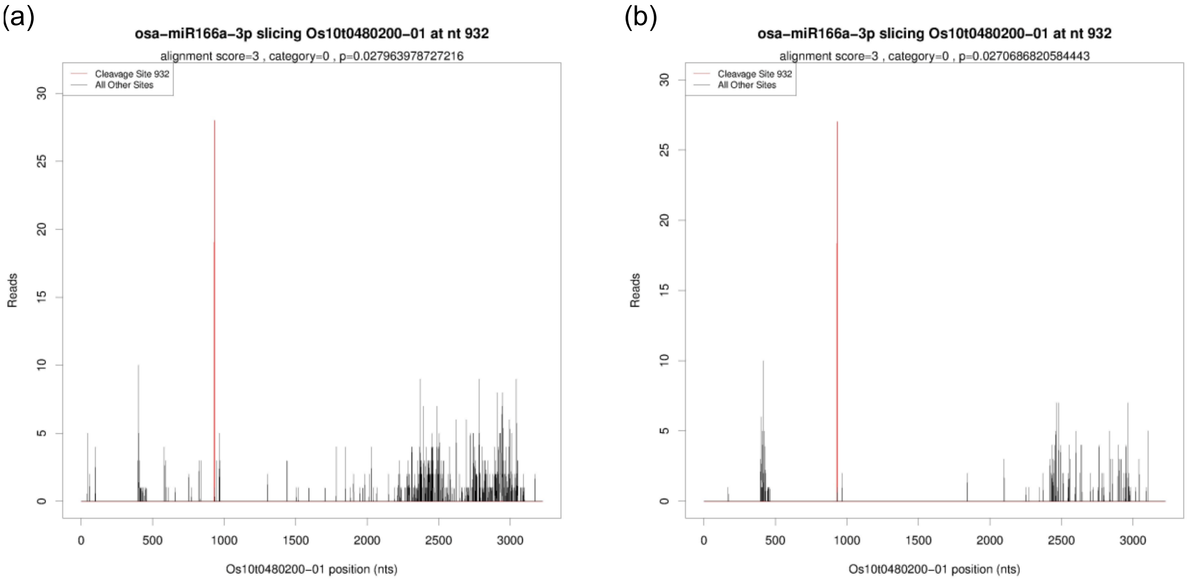

Figure S10 T-plots of miR166a-3p targeting Os10t0480200-01 in (a) gall sample; (b) root tip sample.
